# Lance Adams Syndrome: A Rare Case Presentation of Myoclonus From Chronic Hypoxia Secondary to COVID-19 Infection

**DOI:** 10.7759/cureus.20321

**Published:** 2021-12-10

**Authors:** Rabia Muddassir, Abdelrahman Idris, Noura Alshareef, Ghaidaa Khouj, Rimaz Alassiri

**Affiliations:** 1 Department of Neurology, Internal Medicine, Security Forces Hospital, Makkah, SAU; 2 Department of Medicine and Surgery, Collage of Medicine, Umm Al-Qura University, Makkah, SAU

**Keywords:** chronic, covid-19, hypoxia, myoclonus, lance-adams syndrome

## Abstract

Coronavirus disease 2019 (COVID-19) pandemic is caused by the severe acute respiratory syndrome Coronavirus 2 (SARS-CoV-2) and since the outbreak, many neurological features and syndromes are reported with this multi-organ viral infection. Lance-Adams syndrome (LAS) also referred to as chronic post hypoxic myoclonus is defined as action myoclonus which can occur as generalized, focal, or multifocal repeated myoclonic motor movements which involve the face, trunk, or extremities and it is one of the neurological complications that are related to COVID-19 infection. LAS is reported as a delayed complication of cardiac arrest, which causes cerebral hypoxia leading to myoclonus. We report a case of a 58-year-old male patient diagnosed as a case of LAS secondary to hypoxia occurring because of COVID-19 without cardiac arrest and to the best of our knowledge it is the second case reported with this similar mechanism. Moreover, we discuss the possible pathophysiological relationship between LAS and COVID-19 and various treatment strategies. Eventually, we review the related articles in the literature regarding the LAS and various types of myoclonus associated with COVID-19 infection.

## Introduction

The severe acute respiratory syndrome coronavirus 2 (SARS-CoV-2) was first discovered in Wuhan, China, in December 2019, and the global pandemic of Coronavirus disease 2019 (COVID-19) on May 1, 2021, has reached 153 million cases. Common presentations are more of respiratory symptoms including sore throat, cough, and shortness of breath, along with fever, myalgia, and fatigue. Additionally, neurological manifestations affecting the central and peripheral nervous system have been identified since the outbreak began [1-3]. SARS-CoV-2 can cause central nervous system (CNS) damage in two ways: directly through viral CNS invasion or indirectly through post-infectious disease [4]. The virus attaches to angiotensin-converting enzyme 2 (ACE2) receptors located in the lung, neurological system, and skeletal muscles. Manifestations of CNS involvement are reported in up to 25% of COVID-19 cases. While common non-specific symptoms of CNS include dizziness, headache, and altered mentation, several CNS syndromes including meningoencephalitis, cerebrovascular events, seizures, and CNS neuro-immunological disorders have also been reported [2].

Post-hypoxic myoclonus (PHM) is a neurological complication characterized by uncontrolled myoclonic jerks following cardiac arrest. PHM is divided into two types. The acute type of PHM occurs within 12 hours to 48 hours of hypoxic insult and indicates a poor prognosis. The chronic type of PHM, which is known as Lance-Adams syndrome (LAS), begins within days to weeks after cardiopulmonary resuscitation (CPR) and persists in patients who have recovered consciousness after CPR [5,6].

We present here a case of LAS secondary to hypoxia occurring because of COVID-19 infection.

## Case presentation

A 58-year-old male patient, not known to have any chronic illnesses, presented to the emergency department with repeated vomiting and decreased oral intake for one week, followed by exertional dyspnea and a productive cough along with fever for the last two days. There was no history of headache, loss of consciousness, seizures, or weakness in the limbs. Other systematic reviews were unremarkable. 

On the initial examination, the patient looked alert but unwell, had respiratory distress, and was not able to lie flat on the bed. There was no cyanosis. The vitals were BP of 153/75 mm of Hg, heart rate of 104 beats/min, temperature of 98.6 °F, and respiratory rate of 40 breaths/min with oxygen saturation of 77% on room air which increased to 94% on 15L non-rebreather mask (NRBM). Chest examination showed bilateral diffuse crackles. On neurological examination, the higher mental function was intact with normal cranial nerves. Motor and sensory systems were intact in all limbs with normal deep tendon reflexes. Planter responses were downgoing. There were no cerebellar signs or signs of meningeal irritation. Other systemic examinations were unremarkable.

The laboratory workup of the patient revealed normal hemoglobin, platelets, renal function test, liver function test, and coagulation profile. Abnormal laboratory test results are shown in Table 1.

**Table 1:** Laboratory test results CRP: C-reactive protein, PCT: procalcitonin, LDH: Lactate dehydrogenase

Blood work	Patient test result	Normal range
WBC	12 × 109/L	4.5–11.0 × 109/L
CRP	12.6 mg/L	Normal less than 10 mg/L
Ferritin	1405 ng/mL	20–250 ng/mL
LDH	612 U/L	140–280 U/L
PCT	0.123 ng/ml	Less than 0.05 ng/ml

**Figure 1:**
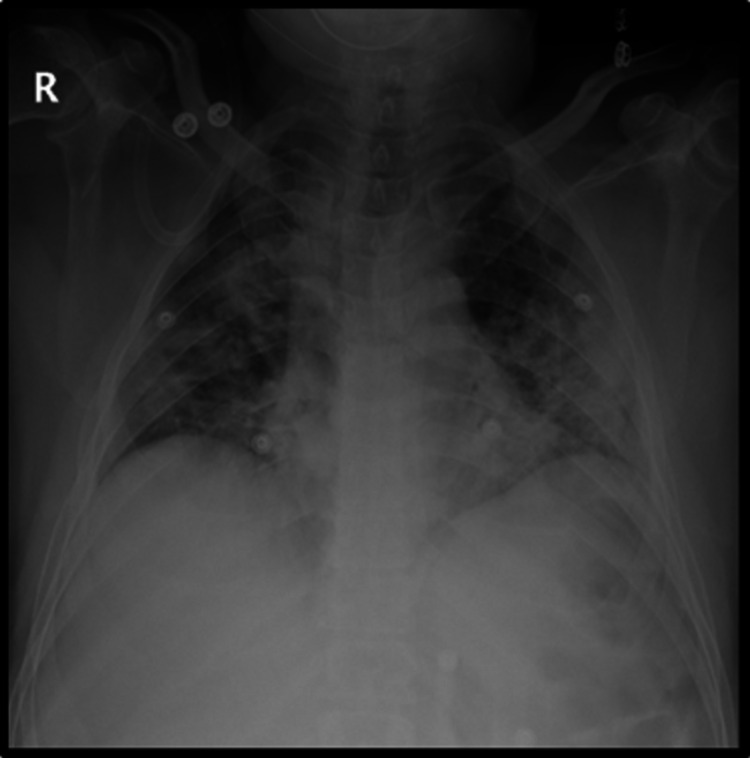
Chest X-ray revealed bilateral non-homogenous opacities at middle and lower lung zones along with blunted both costophrenic angles.

Nasopharyngeal swab test for SARS-CoV-2 by qualitative real-time reverse-transcriptase polymerase chain reaction (rRT-PCR) assay was positive. An electrocardiograph (ECG) showed normal sinus rhythm. Chest X-ray (CXR) revealed bilateral non-homogenous opacities at middle and lower lung zones along with blunted both costophrenic angles (Figure 1).

The patient was isolated and a nasal swab for COVID was taken. The patient’s condition deteriorated six hours after presentation to the hospital. He developed desaturation with an oxygen saturation reaching up to 79% even on 15 liters of NRBM. The patient was transferred to the intensive care unit (ICU) and was shifted on the ventilator. ECG at this time showed sinus tachycardia of 110 beats/minute. He was diagnosed with a case of COVID-19 pneumonia and was started on the specific treatment of COVID 19. During the hospital stay, the patient developed renal failure and was given three sessions of hemodialysis. A week later, the patient was extubated and maintained an oxygen saturation of 94% to 96% on 2 liters of oxygen with a nasal cannula. He was referred to a rehabilitation center.

**Figure 2:**
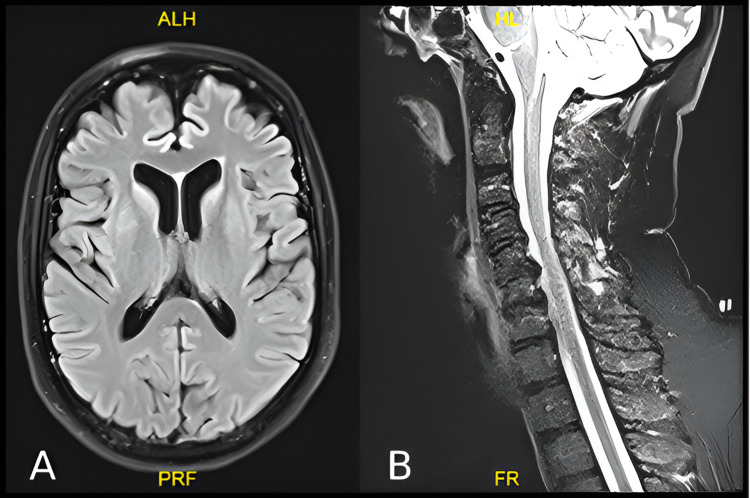
(A) Non-contrast MRI of the brain shows mild age-matched brain involutional changes with no evidence of acute vascular insult. (B) Non-contrast MRI cervical shows multilevel disc lesions with mild neural compression.

**Figure 3:**
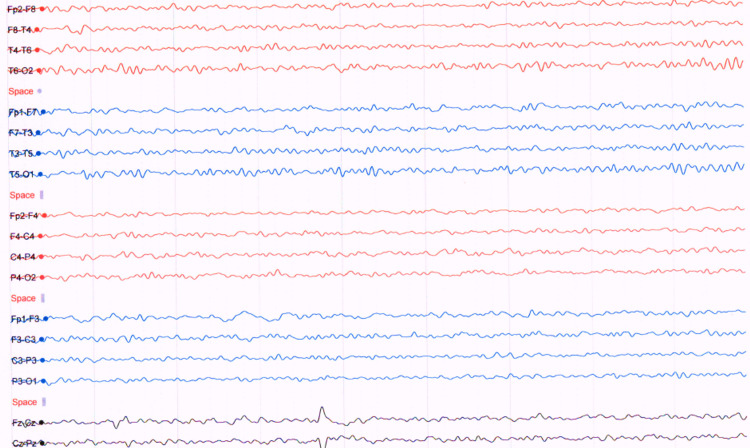
Initial electroencephalogram result showed normal alpha background activity at 8–10 Hz with no frank seizure activity.

**Figure 4:**
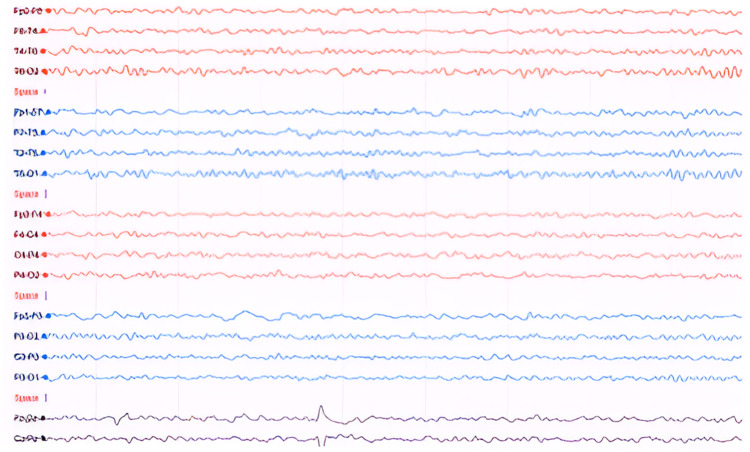
Follow-up EEG after eight weeks result showed normal alpha background activity at 8–10 Hz with no frank seizure activity.

Following six weeks of diagnosis of COVID pneumonia, the patient developed progressive weakness in all four limbs. On examination consciousness was preserved with bilateral mild lower motor neuron facial weakness, generalized areflexia with a power of 3/5 in both proximal and distal muscles in all the limbs. The patient was investigated with magnetic resonance imaging (MRI) brain which showed mild age-matched brain involutional changes with no evidence of acute vascular insult (Figure 2). In addition, an MRI cervical was also performed which showed multilevel disc lesions with mild neural compression (Figure 2). CSF analysis showed a mild increase in proteins with normal cells. The nerve conduction study (NCS) showed axonal motor neuropathy. The patient was suspected to have critical illness neuropathy versus Guillain-Barré syndrome (GBS) and was started on plasma exchange (PE). Following the seven cycles of PE, the weakness in the limbs improved to a power of 4/5 both proximal and distal muscles but the patient started to have postural and action myoclonic tremor mainly in the face and upper arm, distal more than proximal. There were no jerks in the lower limbs. Myoclonic jerks were triggered by touch, sounds, and startles and disappeared with relaxation and sleep. There was no nystagmus or opsoclonus observed in the eyes. Cognitive functions were intact. The patient was able to sit with support, however, was unable to walk. EEG done initially as well as after eight weeks showed normal alpha background activity at 8 to 10 Hz with no frank seizure activity nor encephalopathy (Figures 3-4). With the suspicion of PHM, the patient was started initially on tablet clonazepam 0.25 mg once daily, however, it was not tolerated, so he was switched to tablet sodium valproate 500 mg once daily at bedtime along with tablet levetiracetam 500 mg twice daily. After two weeks of initiating the medication, the frequency of jerks improved and the patient was able to sit unsupported. Following three months, myoclonus has improved, and the patient is able to walk with support.

## Discussion

COVID-19 pandemic is caused by the SARS-CoV-2 coronavirus. Initial studies reported that COVID-19 patients will become critically ill by 5% and will require intensive care. According to the limited evidence available, these patients are more prone to develop neurological problems [7]. Of the above-described neurological complications, myoclonus is one of the recognized unique presentations of COVID-19 which is defined as an uncontrollable involuntary movement characterized by rapid, short, recurrent muscular spasms affecting the face, limbs, and trunk. It can be spontaneous or triggered by various external events including noise, movement, and light [8].

Myoclonus may arise in certain neurological diseases as well as toxic and metabolic disorders including severe hypoxic brain damage [8]. Myoclonus is observed in 20% of cardiac arrest patients within 72 hours of cardiopulmonary resuscitation [9], but less is known about the frequency of myoclonus in patients who experience hypoxia without cardiac arrest. The pathogenesis of myoclonus seen in COVID-19 patients is still uncertain.

As for COVID-19, different types of myoclonus are reported in the literature including generalized myoclonus [10], Opsoclonus-myoclonus syndrome [11], myoclonus with cerebellar ataxia [12], multifocal myoclonus [13], and post-hypoxic myoclonus also known as LAS [14]. LAS is defined as action myoclonus which can occur as generalized, focal, or multifocal repeated myoclonic motor movement [15].

Although the pathophysiology of LAS is poorly understood, the literature suggests that it can originate from subcortical and/or cortical structures [16,17]. In addition, the neurotransmitters associated with LAS are known to be serotonin and gamma-aminobutyric acid (GABA), and GABA may interact with the serotonin system to reduce post-hypoxic myoclonus, and serotonin depletion within the inferior olive nucleus has been believed to play a role [18]. It is important to differentiate LAS from acute post-hypoxic seizures, in which patients remain in a comatose or vegetative state [6].

Our literature research from 2019 to 2021 revealed only one single case of LAS secondary to hypoxemia without cardiac arrest in COVID-19. To the best of our knowledge, we report the second case of LAS due to COVID-19 hypoxemia without cardiac arrest. It is worth mentioning that our patient had a similar presentation of hypoxia but in our case, the oxygen saturation was relatively more severe around 77% as compared to the other case where hypoxia without cardiac arrest reached up to 87% [14]. In addition, our patient had renal failure along with neuropathy which is one of the recognized complications of COVID-19. Although the diffuse involvement of the peripheral nervous system in the form of acute neuropathy is one of the rare complications associated with SARS-CoV-2 coronavirus infection occurring simultaneously or weeks after infection, it is still important to consider this possibility as it requires specific treatment along with conventional management of COVID-19 infection [19].

The MRI of the brain in our case was completely normal. In contrast, MRI in other cases showed brainstem and cortical ischemic lesions [14]. In terms of treatment, the reported case showed improvement to clonazepam which has been shown to raise brain serotonin levels (as well as CSF 5HIAA levels) in therapeutic doses [14]. However, our patient was intolerant to clonazepam, so he was switched to sodium valproate along with levetiracetam to which he remarkably responded with 80% resolution of the myoclonus.

Post-hypoxic myoclonus may be a long-term movement disorder complication of this devastating pandemic and it is difficult to treat [14]. The therapy for LAS is unknown, though it has been suggested that a combination of drugs based on neurotransmitters be used. In a study of more than 100 patients with LAS, Frucht and Fahn discovered that clonazepam, valproate, and piracetam were beneficial in around half of the cases [20].

In summary, the similarity between the previous reported LAS case and this case [14] is the patients were not young, the degree of hypoxia was not severe, and neither of them had a cardiac arrest. In both cases, the onset of action myoclonus was four to six weeks from the onset of hypoxia. There were no opsoclonus, nystagmus, or cognitive changes. EEG and MRI brain were found to be normal in our case, however, MRI brain in the reported case showed some structural brain damage. In addition, it is important to highlight that our patient showed complete recovery from renal failure and had no other metabolic disturbances except initial damage from chronic hypoxia at the beginning which contributed to myoclonus. The outcome in both cases was excellent with remarkable improvement in the myoclonus and total independence of daily activities. In contrast, LAS secondary to cardiac arrest carries a grave prognosis with a lifelong disability.

## Conclusions

In conclusion, despite all the literature research suggesting a poor prognosis of LAS, our patient showed a remarkable improvement to the treatment. One of the possible reasons could be the different etiology of LAS, as it is usually related to hypoxia secondary to cardiopulmonary resuscitation which carries a grave prognosis; however, in our patient, hypoxia was present without any cardiac arrest.

## References

[1] World Health Organization (2020 (2020). Coronavirus disease (COVID-19) pandemic. https://www.who.int/emergencies/diseases/novel-coronavirus-2019.

[2] Mao L, Jin H, Wang M (2020). Neurologic manifestations of hospitalized patients with coronavirus disease 2019 in Wuhan, China. JAMA Neurol.

[3] Cagnazzo F, Arquizan C, Derraz I (2021). Neurological manifestations of patients infected with the SARS-CoV-2: a systematic review of the literature. J Neurol.

[4] Koralnik IJ, Tyler KL (2020). COVID- 19: a global threat to the nervous system. Ann Neurol.

[5] Lance JW, Adams RD (1963). The syndrome of intention or action myoclonus as a sequel to hypoxic encephalopathy. Brain.

[6] English WA, Giffin NJ, Nolan JP (2009). Myoclonus after cardiac arrest: pitfalls in diagnosis and prognosis. Anaesthesia.

[7] Guan WJ, Ni ZY, Hu Y (2020). Clinical characteristics of coronavirus disease 2019 in China. N Engl J Med.

[8] Sutter R, Ristic A, Rüegg S, Fuhr P (2016). Myoclonus in the critically ill: diagnosis, management, and clinical impact. Clin Neurophysiol.

[9] Malhotra S, Mohinder K (2012). Lance-Adams syndrome: Difficulties surrounding diagnosis, prognostication, and treatment after cardiac arrest. Anesth Essays Res.

[10] Rábano-Suárez P, Bermejo-Guerrero L, Méndez-Guerrero A (2020). Generalized myoclonus in COVID-19. Neurology.

[11] Emamikhah M, Babadi M, Mehrabani M (2021). Opsoclonus-myoclonus syndrome, a post-infectious neurologic complication of COVID-19: case series and review of literature. J Neurovirol.

[12] Chan JL, Murphy KA, Sarna JR (2021). Myoclonus and cerebellar ataxia associated with COVID-19: a case report and systematic review. J Neurol.

[13] Latorre A, Rothwell JC (2020). Myoclonus and COVID-19: a challenge for the present, a lesson for the future. Mov Disord Clin Pract.

[14] Ros-Castelló V, Quereda C, López-Sendón J, Corral I (2020). Post-hypoxic myoclonus after COVID-19 infection recovery. Mov Disord Clin Pract.

[15] Freund B, Kaplan PW (2017). Post-hypoxic myoclonus: differentiating benign and malignant etiologies in diagnosis and prognosis. Clin Neurophysiol Pract.

[16] Venkatesan A, Frucht S (2006). Movement disorders after resuscitation from cardiac arrest. Neurol Clin.

[17] Hallett M (2000). Physiology of human posthypoxic myoclonus. Mov Disord.

[18] Matsumoto RR, Truong DD, Nguyen KD, Dang AT, Hoang TT, Vo PQ, Sandroni P (2000). Involvement of GABA(A) receptors in myoclonus. Mov Disord.

[19] Diez-Porras L, Vergés E, Gil F, Vidal MJ, Massons J, Arboix A (2020). Guillain-Barré-Strohl syndrome and COVID-19: case report and literature review. Neuromuscul Disord.

[20] Frucht S, Fahn S (2000). The clinical spectrum of posthypoxic myoclonus. Mov Disord.

